# Original Investigations of Cattle Diseases in Nebraska

**Published:** 1889-12

**Authors:** 


**Original Investigations of Cattle Diseases in Nebraska.** 1886–1888. By FRANK S. BILLINGS, Director of the Patho-Biological Laboratory of the State of Nebraska. Bulletins 7, 8, 9, and 10. Lincoln, Neb. 1889. State Journal Co., Printers.

In a beautiful volume of 267 pages are published the original observations of Dr. Billings on the Diseases of Cattle in Nebraska. This work is volume third of Dr. Billings's investigations, and shows the author to be a careful and painstaking observer.

The patho-biological laboratory of the University of Nebraska was one of the first of its kind to be established in the United States, and from the character of the work which has gone from its walls, it is safe to infer that it has met, if not surpassed, all expectations. All credit is due Dr. Billings, as director of this laboratory, for the excellent work in veterinary and human pathology, which has from time to time appeared in the "Bulletins of the Agricultural Experimental Station of Nebraska." This work has not been so much in the line of confirmatory evidence, in support of theories and facts furnished by other observers, as to take lead in the discovery of facts and creation of theories.

The Southern cattle-plague, or Texas fever, is taken up at some length in this volume, and the bacteriology and pathology of this and yellow fever are carefully studied and compared. The author considers both as extra-organismal infectious septicemia, with a remarkable association of analogies, yet does not consider the two diseases identical.

Article II. treats of "The Corn-Stalk Disease in Cattle." This paper appeared in the July, August and September (1889) numbers of this JOURNAL.

Article III. discusses the history and peculiarities of a new malady, the "So-called Hydroplotra in Cattle," which broke out in some parts of Nebraska in 1886 and 1889. The exact nature of the disease remains unknown.

Article IV. treats of Contagious Inflammation of the Cornea in Cattle. This paper appeared in the April (1889) number of this JOURNAL.

Article V. describes briefly a "Singular Disease of the External Sexual Organs in Cows."

This volume is from the press of the State Journal Co., Lincoln, Neb., and is an excellent specimen of press-work. W. C. K.

